# The transcriptional trajectories of pluripotency and differentiation comprise genes with antithetical architecture and repetitive-element content

**DOI:** 10.1186/s12915-020-00928-8

**Published:** 2021-03-25

**Authors:** Aristeidis G. Telonis, Isidore Rigoutsos

**Affiliations:** 1 Computational Medicine Center, Sidney Kimmel College of Medicine, Thomas Jefferson University, 1020 Locust Street, Suite M81, Philadelphia, PA 19107 USA; 2 Department of Human Genetics, Miller School of Medicine, University of Miami, Miami, FL 33136 USA

**Keywords:** Embryo development, Repetitive elements, Retrotransposons, Genome architecture, Gene length, Exon, Intron, Transcriptional regulation, Tissue specificity, Pyknons

## Abstract

**Background:**

Extensive molecular differences exist between proliferative and differentiated cells. Here, we conduct a meta-analysis of publicly available transcriptomic datasets from preimplantation and differentiation stages examining the architectural properties and content of genes whose abundance changes significantly across developmental time points.

**Results:**

Analysis of preimplantation embryos from human and mouse showed that *short* genes whose introns are *enriched* in Alu (human) and B (mouse) elements, respectively, have higher abundance in the blastocyst compared to the zygote. These highly expressed genes encode ribosomal proteins or metabolic enzymes. On the other hand, *long* genes whose introns are depleted in repetitive elements have lower abundance in the blastocyst and include genes from signaling pathways. Additionally, the sequences of the genes that are differentially expressed between the blastocyst and the zygote contain distinct collections of pyknon motifs that differ between up- and down-regulated genes. Further examination of the genes that participate in the stem cell-specific protein interaction network shows that their introns are *short* and enriched in Alu (human) and B (mouse) elements. As organogenesis progresses, in both human and mouse, we find that the primarily short and repeat-rich expressed genes make way for primarily longer, repeat-poor genes. With that in mind, we used a machine learning-based approach to identify gene signatures able to classify human adult tissues: we find that the most discriminatory genes comprising these signatures have long introns that are repeat-poor and include transcription factors and signaling-cascade genes. The introns of widely expressed genes across human tissues, on the other hand, are short and repeat-rich, and coincide with those with the highest expression at the blastocyst stage.

**Conclusions:**

Protein-coding genes that are characteristic of each trajectory, i.e., proliferation/pluripotency or differentiation, exhibit antithetical biases in their intronic and exonic lengths and in their repetitive-element content. While the respective human and mouse gene signatures are functionally and evolutionarily conserved, their introns and exons are enriched or depleted in *organism-specific* repetitive elements. We posit that these organism-specific repetitive sequences found in exons and introns are used to effect the corresponding genes’ regulation.

**Supplementary Information:**

The online version contains supplementary material available at 10.1186/s12915-020-00928-8.

## Background

Fast accumulating data provide increasing evidence that the genomes of higher organisms contain actionable information that goes well beyond the annotated sequences of protein-coding genes. The architecture of chromosomes, genomic regions, and individual genes as well as their relative orientation and placement can have implications for the dynamics of gene expression. Within this context, evidence has shown that introns are not merely “linkers” of exons [1]. In fact, introns have been shown to be sources of important molecules such as microRNA (miRNA) [2], piRNA [3, 4], and transfer RNA (tRNA) [4] and to maintain functional conservation in the absence of sequence conservation [3]. Introns also provide transcription factor binding sites [5], mark chromatin structures [6], or regulate the production of circular RNA [7, 8]. They have also been found to harbor trait- and disease-associated mutations [9, 10]. Thus, introns can serve as very potent gene regulators [11, 12].

Introns are at the crossroads of evolution and genome complexity [13, 14]. This is highlighted by a growing body of evidence on the importance of intron length and density, from the standpoints of evolution [15–19] and physiology [20, 21]. Highly and/or broadly expressed genes are on average short and compact [21–24]. It has also been observed that stress-response genes have fewer introns [25], presumably reflecting a need for rapid transcription. In fact, shorter exonic and intronic length is correlated with transcriptional and translational speed, a key requirement of rapidly cycling cells [20, 26, 27]. Intuitively, one expects shorter genes to provide fewer opportunities for complex sequence-based regulation and longer genes to be involved in more complex, tissue-specific processes [28–30].

Cell proliferation and differentiation are viewed as polar opposite states at multiple biological levels. Metabolically, rapidly proliferating cells favor aerobic glycolysis; this is true of cancer cells too (Warburg effect) [31]. Transcriptionally, the genes expressed during proliferation exhibit a codon usage bias that is distinct from that of genes that are differentiation-specific; this bias is also evident at the level of tRNA pools in each state [32].

Interestingly, the expression of repetitive elements has also been associated with the stem cell phenotype [33–35], including the pluripotent state of early embryogenesis [36, 37]. However, accumulating evidence suggests that repetitive elements are distributed across the genome in a non-random manner and that their expression is regimented [38–40] and consequential [41–46].

Repetitive sequences have, by definition, multiple instances on the genome. They can be long, well-defined repeats such as the Alu or LINE elements. Or, they can be shorter *k-mers* that appear identically in intronic, exonic, or intergenic sequences. One such category includes the DNA motifs known as pyknons, which we reported previously [40]. Pyknons have at least one copy in messenger RNAs (mRNAs), and many additional intronic and intergenic copies [40], which can be sense or antisense to the mRNAs [3]. The simultaneous presence of pyknons in both exonic and non-exonic sequences suggests their involvement in gene expression regulation [40, 47, 48], something that was recently shown in the context of colon cancer [43, 49].

**Fig. 1:**
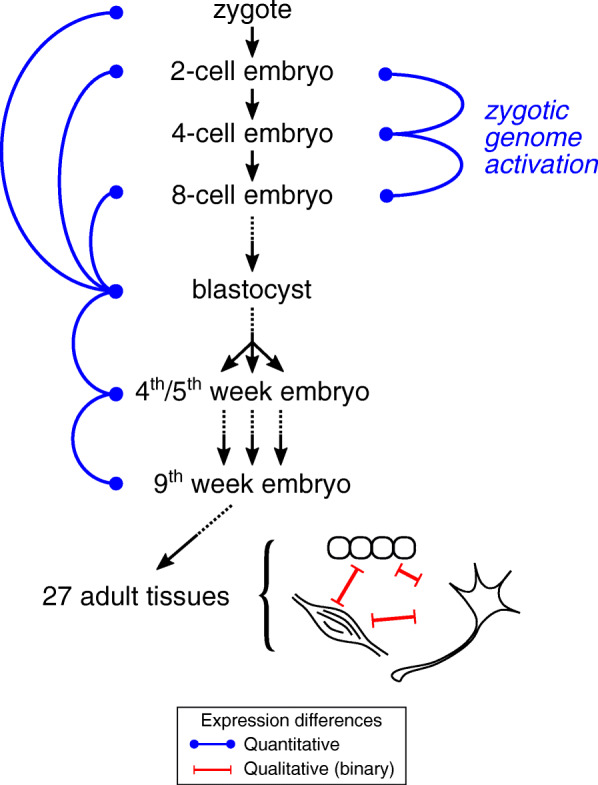
Schematic representation of the major comparisons in this study. We examined how expression profiles change in early and later embryogenesis. Our reference point mainly was the blastocyst stage in both human and mouse, but we also examined zygotic genome activation as well as binary expression differences in fully differentiated adult tissues. Blue lines indicate quantitative comparisons, i.e., differences at expression levels, while red lines indicate qualitative comparisons, i.e., genes expressed or not (on or off) among tissues

Against this background, we sought to determine whether human and mouse genes that are associated with pluripotency and/or a proliferative phenotype exhibit biases in their length or repetitive-element content. To this end, we used publicly available datasets, focusing on elucidating the architecture and sequence content of genes whose abundance changes between proliferation and differentiation (Fig. 1).

## Results

We first analyzed gene expression datasets from preimplantation human [50, 51] and mouse [50, 52] embryos and identified genes that have higher or lower abundance in the blastocyst compared to the zygote, 2-cell, or 8-cell embryo stage. To ensure robustness of the findings, we used two independent datasets for each of human and mouse, respectively. Each dataset was generated using a different quantification methodology (microarray and deep sequencing, respectively). We thresholded and analyzed each of the four datasets separately and found the results to be reproducible (Additional file 1: Supplemental Figure S1). Out of an average of 12,015 genes in each dataset, we found 2709 statistically significantly up-regulated and 5286 down-regulated genes in the blastocyst with respect to earlier developmental time points (false discovery rate, FDR ≤ 5%; Additional file 2: Supplemental Table S1). Among the genes that are more abundant in the blastocyst are mitochondrial membrane transports (e.g., *TOMM6* and *TIMM13*), glutathione metabolism genes (e.g., *GPX4*, *GSTP1*, and *GSTO1*), ribosomal proteins (e.g., *RPL4* and *RPL6*), and metabolic genes (e.g., *HK1*, *IDH3B*, and *TKT*). On the other hand, notable genes among those with lower abundance in the blastocyst include *NCOA1*, *AK5*, *GRK5*, *ITGA9*, and *CLOCK*. Examining the associated pathways, we found that ribosome, glycolysis, citric acid cycle, and oxidative phosphorylation are enriched among the genes that are more abundant in the blastocyst (Additional file 2: Supplemental Table S1). On the other hand, signaling pathways (including MAPK, cAMP, JAK-STAT, and Wnt) are enriched among the genes that are more abundant in the zygote (Additional file 2: Supplemental Table S1). These results are in agreement with previous studies [26, 53] and provide a robust dataset for further mining.

### Biases in length and repetitive-element content among expressed genes change monotonically with the preimplantation developmental stage

In zebrafish, the genes that are expressed during the transition from the zygote to a highly proliferative population of cells exhibit length biases [26]. We hypothesized that a similar bias may characterize human and mouse genes as well [21, 22].

**Fig. 2:**
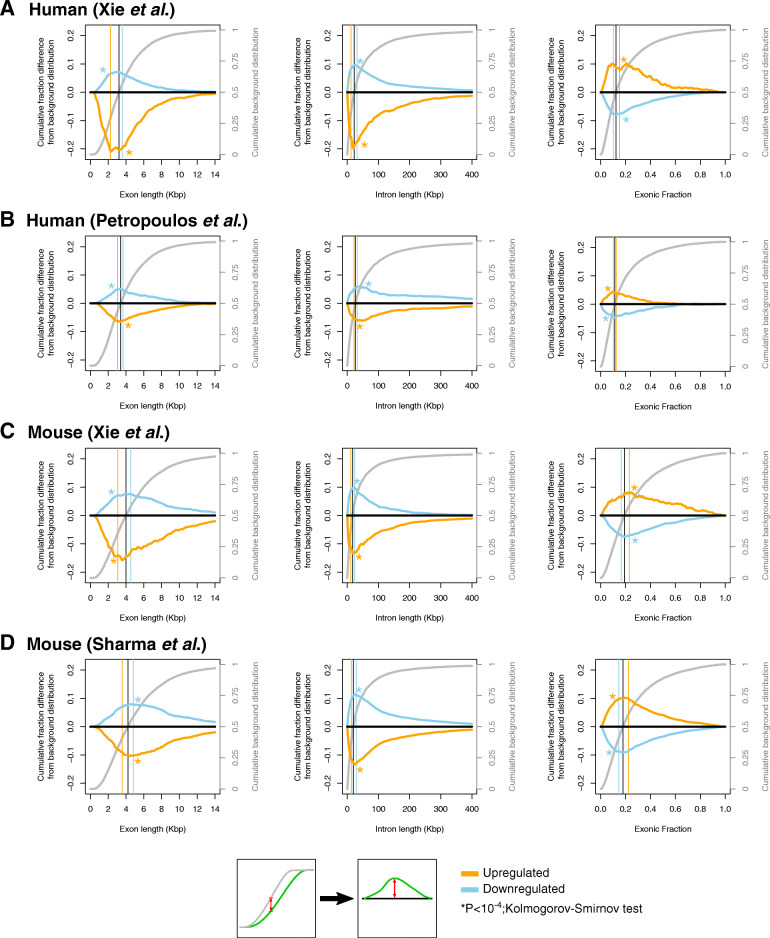
Gene length and compactness biases in gene expression changes during preimplantation development. Exon (left panel) and intron (middle panel) length and exonic content (right panel) distributions of genes that are up-regulated (orange curves) or down-regulated (cyan curves) in blastocyst as compared to early embryo in human (**a**, **b**) and mouse (**c**, **d**) in comparison to the background genes (black curves). As shown in the panels, the primary *Y* axis describes the difference of each cumulative distribution from the background cumulative distribution (curves are smoothened with a 3-point moving average); the background cumulative distribution is plotted in gray line projecting on the secondary *Y* axis. Positive values reflect an increase in each parameter, e.g., a shift of the distribution towards longer exons. Vertical lines are drawn at the median value of each gene set. Asterisks indicate a statistically significant difference from the background distribution (*P* value < 10^−4^; Kolmogorov-Smirnov test)

We computed the distributions of the exonic and intronic lengths in nucleotides (nts) for the genes that are differentially abundant between the blastocyst and earlier embryonic stages, i.e., the zygote, 2-cell, or 8-cell embryo depending on the study (see the “Materials and methods” section; Additional file 3: Supplemental Table S2), and juxtaposed them to the respective length distributions of all expressed genes in each dataset (background). We found that genes with *higher* abundance in the blastocyst compared to earlier embryonic time points have significantly *shorter* exons and introns (*P* value < 10^−4^; Kolmogorov-Smirnov test). On the other hand, genes with *lower* abundance in the blastocyst have significantly *longer* exons and introns (*P* value < 10^−4^; Kolmogorov-Smirnov test). These observations hold true for both human (Fig. 2a, b) and mouse (Fig. 2c, d) embryos.

In addition to being shorter, the genes with higher abundance in the blastocyst compared to respective earlier embryo stages had more of their genomic span occupied by exons (*P*  value  < 10^−4^; Kolmogorov-Smirnov test). Notably, the opposite holds true for genes whose abundance is lower in the blastocyst compared to the respective earlier embryo stages (right panels; Fig. 2a–d).

We note that these observations remain unchanged even when we form the background distribution by considering *all* human or mouse protein-coding genes (Additional file 1: Supplemental Figure S1B-C).

**Fig. 3:**
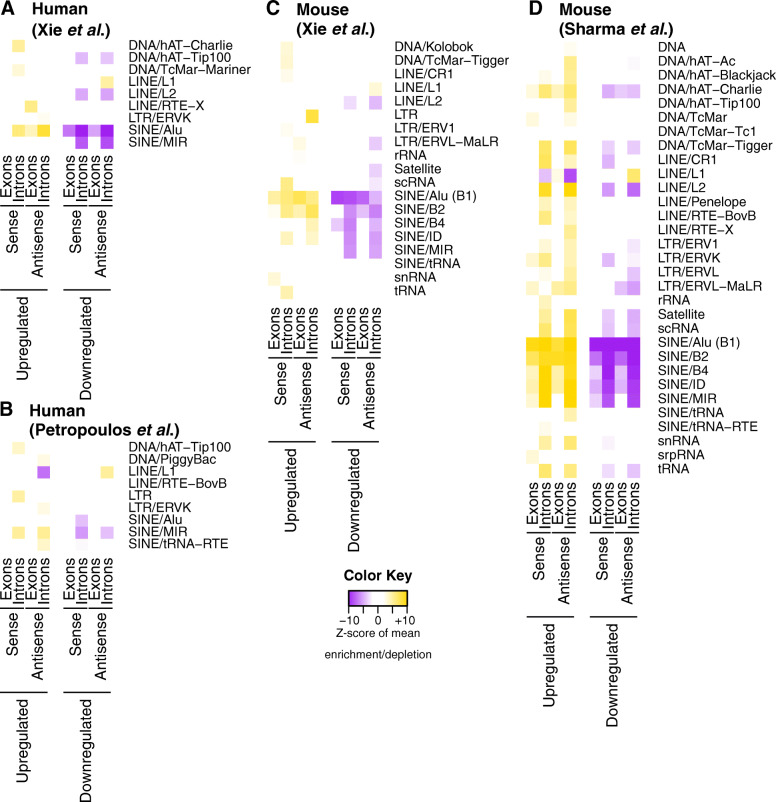
Repetitive-element density biases in gene expression changes during preimplantation development. Heatmaps of enrichment and depletion scores of the repetitive density in genes that are up-regulated or down-regulated in the blastocyst as compared to early embryo in human (**a**, **b**) and mouse (**c**, **d**) embryos. The heatmaps visualize the *Z*-score of the observed mean density in each repetitive-element family with respect to an expected distribution constructed with Monte Carlo simulations. A positive *Z*-score (colored orange) represents a density more than expected by chance, while a negative *Z*-score (colored purple) shows that the observed density is lower than expected. *Z*-scores were computed independently for intron and exons and for the sense and antisense orientation of these genomic regions. For each panel (**a**–**d**), only the repetitive families with at least one significant enrichment or depletion (absolute *Z*-score larger than 2) are shown. Additional file 4: Supplemental Table S3 includes the values used for plotting the heatmaps as well as the respective FDR scores

The differences in the exonic and intronic lengths of those two groups of genes prompted us to also examine their nucleotide composition for other possible biases. In particular, we investigated whether the introns and exons of the genes that are up-regulated or down-regulated in the blastocyst are enriched or depleted in any families of repetitive elements. We used Monte Carlo simulations (see the “Materials and methods” section), distinguishing between sense and antisense instances of repetitive elements with respect to the orientation of the genes at hand. For this analysis, we calculated “repetitive-element content per unit length” in order to account for the fact that different genes have different lengths (see the “Materials and methods” section for more details). In Fig. 3, we show heatmaps of the *Z*-scores that capture the calculated enrichments and depletions with respect to a random-generated background distribution: in all instances, the corresponding FDR value is ≤ 5%. Additional file 4: Supplemental Table S3 lists the various *Z*-scores and associated FDR values.

Figure 3 makes it strikingly evident that the genes that have higher abundance in the blastocyst compared to respective earlier embryonic stages are also denser in repetitive elements than would have been expected by chance. On the other hand, the genes that have lower abundance in the blastocyst are depleted in repetitive elements. This observation holds true for both exons and introns in human (Fig. 3a, b) and mouse (Fig. 3c, d), and for both orientations of the repeats with regard to the genes' sequences. Of note, introns are enriched or depleted in more categories of repetitive elements than exons.

The repetitive elements whose sequences are over- or under-represented in the examined sequences include DNA transposons, Long Terminal Repeats (LTR), short interspersed nuclear elements (SINE), and the L1 category of long interspersed nuclear elements (LINE). SINE elements are most enriched among the genes whose abundance is higher in the blastocyst, in both humans (Alu, MIR) and mice (B elements, MIR) and with *Z*-scores as high as + 10.1 (FDR < 10^−13^). On the other hand, SINE and other repeat categories are *depleted* among the genes whose abundance is lower in the blastocyst, with *Z*-scores as low as − 13.0 (FDR < 10^−19^).

The L1 category represents an exception in the above observations. This is best exemplified by the mouse dataset described in Fig. 3d. As can be seen, the introns of the genes with higher abundance in the blastocyst are *depleted* in both sense and antisense L1 elements (average *Z*-score of -6.4; FDR < 5%) whereas the introns of the genes with low abundance in the blastocyst are *enriched* in antisense L1 elements (average *Z*-score = + 5.2; FDR < 5%).

One important characteristic of the developmental stages studied here is zygotic genome activation (ZGA) [54]. It is conceivable that the observed differences in transcript composition, transcript length, and repetitive-element biases might be associated with transcripts transcribed de novo after ZGA. To examine this possibility, we focused on the human and mouse datasets of Xie et al. [50]. Specifically, and for different time points for mouse and human embryos, we identified the genes that are up-regulated as the zygotic genome is activated (Additional file 2: Supplemental Table S1) [54]. We found that both the exons and the introns of the corresponding sets of genes are shorter than the background gene population (*P*  value  < 10^−4^; Kolmogorov-Smirnov test) and are enriched in the same repetitive-element families shown in Fig. 3 (Additional file 1: Supplemental Figure S2A-B; Additional file 4: Supplemental Table S3). Moreover, we found that the ZGA-related genes overlap significantly with the genes that have higher abundance in the blastocyst (*P* value < 10^−4^; hypergeometric test)—see Additional file 1: Supplemental Figure S2C. This indicates that ZGA follows the same architectural patterns but is only part of the transition from the zygote to the blastocyst.

Collectively, the above results suggest that the genes that are expressed during the preimplantation embryogenesis, including ZGA, exhibit specific patterns in terms of gene architecture and sequence content.

### Examples of protein-coding genes having conspicuous overlaps with repetitive elements

The human hexokinase 1 gene, *HK1*, is located on chromosome 10 where it spans ~ 132 kilobases (Kb). Its exonic length is ~ 4.5 Kb, representing 3% of the gene’s total span. Those of the Alu sequences that are sense to this gene are located solely in its introns and span a grand total of ~ 17 Kb. An *additional* 16 Kb of Alu sequences are antisense to this gene’s span. In other words, almost one fourth of *HK1*’s genomic span contains Alu sequences, either in sense or in antisense orientation. Similar observations can be made for the mouse orthologue *Hk1*: its overlap with B elements, in either sense or antisense orientation, is ~ 19%. The density of MIR elements is also consistent in this gene between the two organisms. Approximately 4% of the human orthologue and 7% of the mouse orthologue correspond to MIR sequences in either sense or antisense. Similar observations can be made for *TKT*, *RPL14*, and *KRT8* as well, all of which are differentially abundant and part of the enriched pathways that include metabolism and the ribosome (Additional file 2: Supplemental Table S1).

These examples point to the considerable size of the overlap of repetitive elements on genes and hint to potentially consequential associations at the intersection of genomic architecture, evolution, and developmental stage. We examine these matters and their ramification further in the “Discussion” section.

### The up-regulated and down-regulated genes contain unique pyknon signatures while the pyknons they have in common correspond to SINE/Alu elements

To obtain a more detailed perspective on the extent of sequence similarities between the two groups of genes with opposite expression behavior, we examined their pyknon composition from a qualitative perspective (Additional file 3: Supplemental Table S2). Pyknons are present in virtually all mRNAs [40], overlap with repetitive elements [47], and have been shown to be functionally active in several contexts [43, 48, 49]. As they are short in length, they can be used to conduct more granular analyses than would have been possible using the repetitive elements of RepeatMasker.

**Fig. 4:**
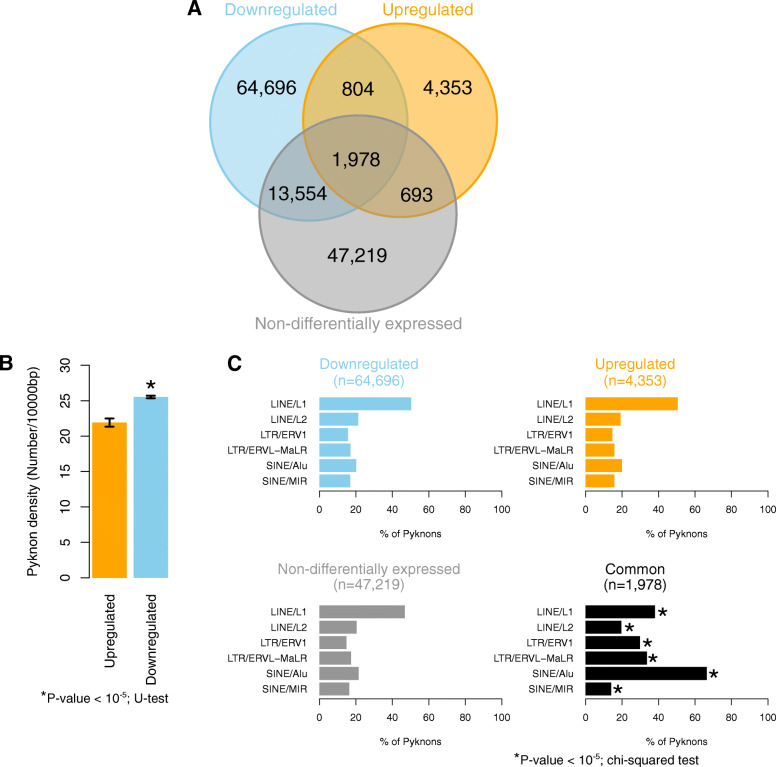
Pyknon content and density biases in gene expression changes during preimplantation development. The results in this figure are from the differential expression analysis of the human embryos from the Xie et al. study of Fig. 1a. **a** Venn diagram of the number of distinct pyknons within the exons of human genes that are up-regulated, down-regulated or non-differentially expressed in the blastocyst compared to the zygote. **b** Number of instances (shown as mean ± standard error) of pyknons per 1000 base pairs in the exons or introns of up-regulated and down-regulated genes. The pyknons in this analysis are from the unique portions of the Venn diagram of **a**. Asterisk indicates statistical significance (*P* values < 0.05; Mann-Whitney *U* test). **c** Barplots showing the percentage of pyknons that overlap with repetitive elements on the whole genome. The asterisks indicate that statistically significant differences exist between the distribution of pyknons of the intersection in repetitive elements in comparison to all three (up-regulated, down-regulated, or non-differentially expressed) of the respective distributions of pyknons that are unique in each gene set (*P* value < 10^−5^; chi-squared test)

We identified 7828 distinct pyknons that overlap the exons of genes that are up-regulated in human blastocyst and 81,032 ones in genes that are down-regulated; 2782 pyknons are shared by the two gene sets (Fig. 4a and Additional file 2: Supplemental Table S1). We found that there are 4353 and 64,696 pyknons uniquely present in the exon spans of up- and down-regulated genes, respectively (Fig. 4a). More than 90% of the genes in each of the up- or down-regulated gene sets contain at least one pyknon. The exons of the down-regulated genes have a higher density in pyknons (Fig. 4b). On the other hand, there is no appreciable difference in the pyknon density of the introns of the up-regulated and down-regulated genes (data not shown).

As pyknons are by definition repeated motifs on the human genome, we examined how they related to the repetitive-element families and distribution biases of Fig. 3a. Specifically, for the pyknons that are unique to the down-regulated and to the up-regulated genes, respectively, we collected all their genomic instances and intersected them with the known repetitive elements. We then calculated the frequency by which they appeared within each type of repetitive element. For the pyknons that are unique to either the up-regulated or the down-regulated genes, or only in the non-DE genes, most of them (~ 45%) can be found within LINE/L1 elements of the genome whereas a smaller proportion (~ 20%) overlapped with SINE/Alu elements (Fig. 4b). Interestingly, the pyknons that were common in all three gene groups mostly overlapped with SINE/Alu (67% of the pyknons). ERV elements were also significantly enriched (Fig. 4b).

Collectively, this analysis positions pyknons as sequence markers for how the gene will change expression during preimplantation development. These findings also suggest that not all members of a family of repetitive elements are equal in this regard: evidently, the pyknons can effectively partition known families of repeats into subsets each of which is associated with the down-regulated genes, up-regulated genes, and unchanged genes, respectively. The findings further support logical connections—presumably ones that capture regulatory events in nature—between repetitive elements and mRNAs that are expressed in early embryogenesis.

### The architecture of early-expressed genes mirrors that of genes comprising the stem cell signature

Above, we showed that early-expressed genes have specific architectural characteristics. The transition from the zygote to the blastocyst can be viewed as the onset of a proliferative phenotype and, for a portion of the cells of the blastocyst, the establishment of a stem cell identity [55]. Considering the latter, we hypothesized that the genes whose abundance is higher in the blastocyst as compared to subsequent embryonic stages may be part of the known stem cell expression signatures.

To test this hypothesis, we downloaded and analyzed the genes involved in the PluriNet protein-protein interaction network [56]. This network is shared among pluripotent stem cells and was generated based on a multitude of stem cell samples. We note that we examined the PluriNet genes with reference to all human protein-coding genes, independent of the genes’ levels of expression at the blastocyst stage.

**Fig. 5:**
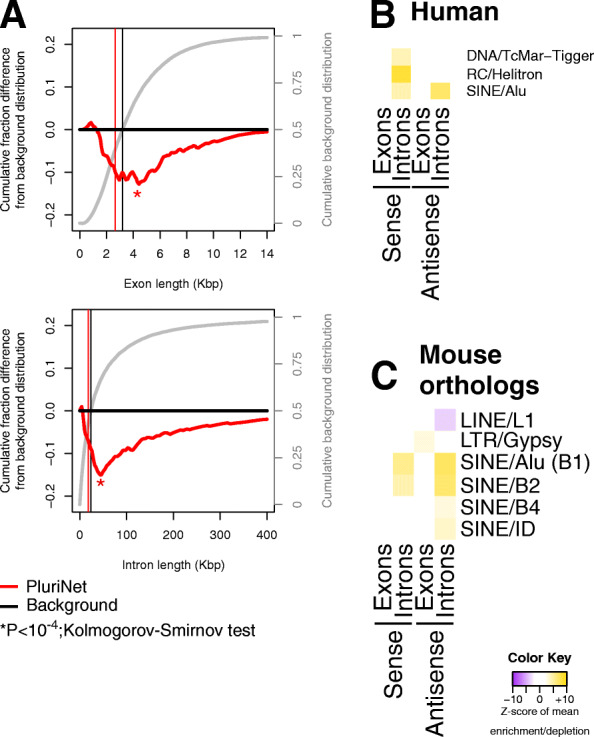
The genes of the stem cell-specific PluriNet network are shorter with organism-specific biases in repetitive densities. **a** Exon (top panel) and intron (bottom panel) length biases. The red curve shows the difference between the cumulative distribution of the PluriNet genes from the background distribution of all human protein-coding genes, plotted as in Fig. 1. Asterisks indicate a statistically significant difference (*P* value < 10^−4^; Kolmogorov-Smirnov test). **b**, **c** Heatmaps of enrichment and depletion scores of the repetitive density in the intronic regions of the PluriNet genes in human (**b**) and of the orthologous genes in mice (**c**)

We found that the genes forming the PluriNet network have shorter lengths (Fig. 5a). Specifically, both exons (top panel of Fig. 5a) and introns (bottom panel of Fig. 5a) are statistically significantly shorter than the background population of human protein-coding genes (*P* value < 10^−4^; Kolmogorov-Smirnov test). When we examined the exons of these genes, we did not find any repetitive-element family to be enriched or depleted. However, when we examined the introns of these genes, we found them to be enriched in sequences from DNA repeats, Helitron and Alu elements (FDR < 5%; Fig. 5b; Additional file 4: Supplemental Table S3).

Next, we identified the mouse orthologues of these genes and examined their overlap with mouse repetitive elements. Exons were again found to lack any notable attributes. However, introns exhibit significant biases (Fig. 5c). Specifically, the intronic regions of the mouse orthologues of the PluriNet signature are significantly denser in B1 and B2 SINE elements in both sense and antisense orientations (FDR < 5%; Fig. 5c; Additional file 4: Supplemental Table S3). We note that L1 elements show an inverse behavior and are depleted in the introns of these genes (FDR < 5%; Fig. 5c; Additional file 4: Supplemental Table S3).

These results parallel the above observations from early development and support the view that the genes that form the signature of a stem cell phenotype have specific structure and content in both human and mouse.

### Gene expression trajectories of differentiation and organogenesis involve longer genes that are less dense in repetitive elements

Having observed that the state of pluripotency is characterized by shorter genes whose exons are enriched in repetitive sequences, we examined whether repetitive elements differ in cells of different lineages or in differentiating cells, and whether lineage-specific genes share common characteristics.

**Fig. 6:**
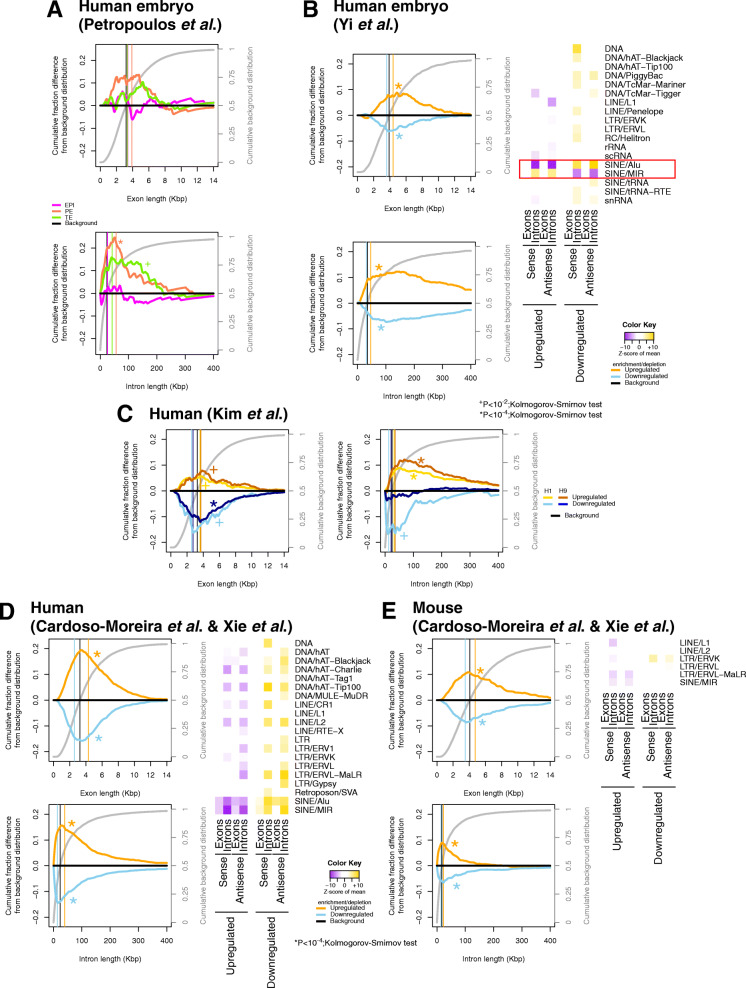
Gene trajectories during differentiation result in overall depletions in repeats. **a** Exon (top) and intron (bottom) distributions of the lineage-specific genes in the human blastocyst. **b** Exon (top-left panel) and intron (bottom-left panel) length biases and heatmap of enrichment and depletion scores (right panel) in up-regulated and down-regulated genes during human organogenesis. **c** Exon (left) and intron (right) length biases in genes that are up-regulated and down-regulated in differentiated embryoid bodies compared to H1/H9 cells. **d**, **e** Length biases in exons (top-left panel) and introns (bottom-left panel), and heatmap of enrichment/depletion scores (right panel) in up-regulated and down-regulated genes during human (**d**) or mouse (**e**) development. The genes included in this analysis are found up- or down-regulated in all seven developing tissues from the Cardoso-Moreira et al. study as compared to the blastocyst from Xie et al. Observations are based on comparisons of rank normalized genes (see text). Asterisks and crosses indicate a statistically significant difference from the background distribution (*P* value < 10^−4^ for an asterisk; *P* value < 10^−2^ for a cross; Kolmogorov-Smirnov test)

We first analyzed the blastocyst lineage signatures that were defined by the Petropoulos et al. study [51]. We examined the lineage-specific genes that the study reported for trophoectoderm (TE), primitive endoderm (PE), and epiblast (EPI), respectively. We found that the genes in PE and TE had significantly longer introns (*P* value < 10^−2^; Kolmogorov-Smirnov test) but not exons (Fig. 6a). The introns of the PE-specific genes exhibited a stronger length bias than the exons. The weaker *P* values in the case of TE could be explained by the relatively low number of genes. Moreover, the PE-specific genes were depleted in Alu elements (Additional file 4: Supplemental Table S3). While limited, these results provide independent support of our findings on gene length and lineage-specific genes.

We then analyzed genes from human embryo at the stage of organogenesis [57], specifically, genes whose expression significantly changes by the 9th week as compared to the 4th week. We found that the genes whose abundance increases during organogenesis have both long exons and long introns (*P* value < 10^−4^; Kolmogorov-Smirnov test; orange curves on Fig. 6a). On the other hand, the exons and introns of genes whose abundance decreases during organogenesis are on average shorter (*P* value < 10^−4^; Kolmogorov-Smirnov test; blue curves on Fig. 6b). When we examined the repetitive-element density of these genes, we observed significant trends. Genes with decreasing abundance during organogenesis are enriched in repetitive sequences; on the other hand, genes with increasing abundance during organogenesis are depleted in repetitive sequences (FDR ≤ 5%; heatmap of Fig. 6b; Additional file 4: Supplemental Table S3). Specifically for Alu elements, the introns of the genes whose abundance increases during organogenesis were significantly sparser in both sense and antisense instances of Alu sequences. Inversely, the introns of the genes whose abundance decreases during organogenesis are significantly denser in Alu sequences. However, the MIR elements, and to a lesser extent the LINE/L1 elements, show the opposite trend (Fig. 6b; Additional file 4: Supplemental Table S3). MIR and Alu elements are highlighted with a red rectangle on Fig. 6b.

We further looked into differentiation, we studied the cases of H1 and H9 human embryonic stem cells forming differentiated embryoid bodies in culture [58] and identified those genes whose abundance changes between the differentiated embryoid bodies and undifferentiated stem cells (Additional files 2 and 3: Supplemental Tables S1 and S2). In both H1 and H9 cells, we found significant biases in the lengths of genes that change in abundance during differentiation (Fig. 6c): the exons of genes whose abundance decreases (resp., increases) with differentiation are significantly shorter (resp., longer) than the background population of genes (*P* value < 10^−4^; Kolmogorov-Smirnov test). Similar observations can be made for the introns of the H1 cells (Fig. 6c). For H9 cells, it is only the introns of up-regulated genes that were statistically significantly different (*P* value < 10^−4^; Kolmogorov-Smirnov test; Fig. 6c). We note that the stronger statistical differences are found in the introns of the differentially expressed genes. In terms of repetitive-element content, the introns of H1 and H9 genes whose abundance increases with differentiation had strong and statistically significant depletion in Alu element density (*Z* score < − 10; FDR < 5%; Additional file 4: Supplemental Table S3).

To examine the differentiation process in more detail, we integrated the data from Xie et al. [50] with the ones from Cardoso-Moreira et al. [59]. The latter dataset includes expression values from seven different tissues from multiple developmental time points in both human and mouse. These datasets were obtained from different laboratories using distinct platforms (microarrays and RNA-sequencing). Consequently, the expression values of genes are not directly comparable without proper normalization. For instance, normalizing to housekeeping genes will produce erroneous results because the expression of ribosomal and metabolic genes, like mouse *Gapdh*, changes during development (see Additional files 2 and 3: Supplemental Tables S1 and S2). To overcome this limitation, we rank-normalized the datasets and considered as differentially abundant those genes whose ranking differed significantly between the compared datasets (see the “Materials and methods” section). Among these differentially ranked genes, the vast majority were common in all seven tissues, in both human and mouse (Additional File 1: Supplemental Figure S3 a-d; Additional File 2: Supplemental Table S1). However, there were tissue-specific gene changes, like the unique upregulation of prothrombin in the liver or the upregulation of Gene Ontology terms related with cardiac muscle development in the heart (Additional File 2: Supplemental Table S1).

We examined the architecture of those genes whose expression differed between the blastocyst and all seven tissues. The genes that were more abundant in the developing tissues as compared to the blastocyst had longer exons and introns in both human and mouse (P-value < 10^-2^; Kolmogorov-Smirnov test; Fig. 6d, e). On the contrary, the genes with lower abundance in the seven tissues had shorter introns and exons in human (P-value < 10^-4^; Kolmogorov-Smirnov test; Fig. 6d, e). 

We also analyzed the repetitive-element content of the differentially expressed genes. In humans, we found a global bias in the density of repeats: the genes with higher abundance in all developing tissues were significantly sparser in repetitive elements whereas those with lower abundance were significantly richer in repeats (Fig. 6d; Additional File 4: Supplemental Table S3). In mouse, the differentially abundant genes exhibited less of a repetitive element bias compared to human (Fig. 6e; Additional File 4: Supplemental Table S3).

Collectively, the results of the previous sections and those shown in Fig. 6 suggest that differentiation follows a trajectory that is essentially the opposite to the one followed when establishing a proliferative/pluripotent phenotype (Fig. 3). At the same time, the case of Alu and MIR elements (Fig. 6b) indicates that the process of differentiation, as captured in Fig. 6a–c, is more complex than merely the inverse of establishing the pluripotency state (Figs. 2 and 3).

### In differentiated tissues, tissue-specific genes are longer and repeat-depleted whereas ubiquitously expressed genes are shorter and repeat-enriched

Our results so far refer to differentiating cells during embryogenesis and do not necessarily describe the attributes of differentiated cells. Thus, we investigated whether length and repeat-element biases exist in differentiated tissues such as those found in the Genotype-Tissue Expression (GTEx) repository [60]. Specifically, we investigated the possibility of such biases in genes that are specific to each tissue.

**Fig. 7:**
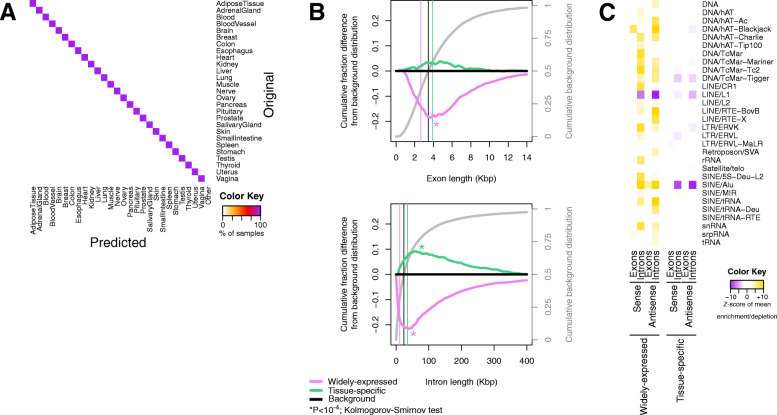
Tissue-specific gene signature and widely expressed genes exhibit opposite length and Alu density biases. **a** Classification of the GTEx cohort using the SVM model trained on the tissue-specific genes. The rows of the heatmap show the original tissue of origin and the columns the predicted tissue type. The color shade of each cell indicates the percentage of samples that were of the “original” respective tissue and were “predicted” to be the respective tissue type. The “Other” category captures samples with low prediction probability. The high percentages on the diagonal indicate the high accuracy of the model. As the SVM was run with 10-fold cross-validation, the heatmap indicates the average of 10 runs. **b** Exon (top) and intron (bottom) length distributions of tissue-specific and widely expressed genes. Asterisks indicate a statistically significant difference from the background distribution (*P* value < 10^−4^; Kolmogorov-Smirnov test). **c** Heatmap showing the enrichment and depletion in repetitive-element families of the widely expressed and tissue-specific genes

We formed tissue-specific gene signatures using our previously developed machine learning approach for extracting models from “binary” expression profiles [61]. In these profiles, each gene is labeled as “expressed” or “not expressed” in a dataset based on whether its abundance exceeds a stringent threshold (Additional file 5: Supplemental Table S4). We demonstrated previously that this methodology can distinguish among 32 different cancer types (from different tissues) [61]. Additionally, the methodology allows us to identify the transcripts with the most discriminatory power [61]. We applied this scheme to the GTEx cohort and found 1505 tissue-specific genes that can discriminate among the 27 normal tissues (Fig. 7a) and also 1340 widely expressed genes, i.e., genes found expressed across all tissues (see the “Materials and methods” section; Additional file 1: Supplemental Figure S4; Additional files 1 and 5: Supplemental Tables S1 and S4).

We compared the tissue-specific and the widely expressed genes from the standpoint of length and sequence biases. The widely expressed genes are enriched in the housekeeping pathways that we found to be abundant in the blastocyst, including the ribosome, oxidative phosphorylation, the citric acid cycle, and spliceosome (Additional file 2: Supplemental Table S1). On the other hand, the genes that comprise the tissue-specific signatures are significantly enriched in homeobox-containing genes and signaling and developmental processes (Additional files 2 and 4: Supplemental Tables S1 and S3; Additional file 1: Supplemental Figure S5). Intriguingly, nine of the 10 most important genes are transcription factors (*TBX15*, *FOXF1*, *TWIST1*, and six HOX genes), whereas the tenth is the kinase-encoding gene *SKAP2* (Additional file 5: Supplemental Table S4).

The length characteristics of the widely expressed and tissue-specific groups of genes exhibit opposite trends. The widely expressed genes have significantly shorter exons and introns. The tissue-specific genes have significantly longer introns (*P* value < 10^−4^; Kolmogorov-Smirnov test). See also Fig. 7b, c and Additional file 4: Supplemental Table S3.

The repetitive-element content of these two groups also exhibits opposite trends. The introns of the widely expressed genes are strikingly enriched in repetitive elements, particularly Alu’s, in both sense and antisense orientations (Fig. 7c). The LINE/L1 category was again a noteworthy exception: the introns are significantly depleted in L1 elements. On the other hand, the tissue-specific gene sets are depleted in repetitive elements but in comparatively fewer categories. We note that, again, SINE/Alu elements exhibit the greatest depletions (Fig. 7c; Additional file 4: Supplemental Table S3).

Collectively, the dichotomy we observe between widely expressed and tissue-specific genes regarding their length biases and repetitive-element content mirrors what we observed in previous results (Figs. 2, 3, and 5): the genes with higher expression in a pluripotent/proliferative state are shorter, repetitive-element rich and represent pathways that are often considered as housekeeping. In contrast, gene sets that establish tissue identity have longer introns, on average; are repetitive-element sparse; and include signaling and transcription factor processes.

## Discussion

In this study, we used publicly available datasets to understand the architecture and repeat content of the human and mouse genes whose abundance changes significantly (a) during early development and (b) during differentiation (Fig. 1). We find that the establishment of pluripotency during the preimplantation period (Figs. 2 and 3) is characterized by the up-regulation of short and compact genes that are markedly dense in diverse types of repetitive elements. On the other hand, genes that are down-regulated during the preimplantation period, or up-regulated during differentiation, are generally longer and less dense in repeats. The very same properties hold true for the genes comprising the previously established stem cell-specific interaction networks (Fig. 5). Additionally, our results suggest that cell type- and potentially tissue type-specific signatures comprise genes whose exons and introns are enriched or depleted in specific categories of repetitive elements (Figs. 6 and 7).

Many of the genes whose abundance increases during preimplantation can be thought of as “housekeeping” genes. This is concordant with previous findings: e.g., Boroviak et al. observed that metabolic pathways and transcriptional, splicing, and RNA transport processes are conserved in mammalian zygotes [53]. Our results suggest that despite the conservation in the pathways per se, the mechanisms by which they are regulated in preimplantation development may not be conserved.

It is important to note that such studies as well as the gene expression datasets we used in this study do not easily discriminate between maternally deposited and embryo-synthesized transcripts. However, an approximation of the genes transcribed de novo at ZGA supports our findings and hints at globally coordinated gene expression programs that show a strong coupling to genes with specific genomic architecture. Indeed, Heyn et al. showed in zebrafish that the first zygotically transcribed genes are short and intron-poor [26]. Our analyses show that the human and mouse genes that are expressed in the early embryo also have short exons and introns. Therefore, it seems reasonable to posit that this is a more general property that holds across vertebrates (and possibly invertebrates) and that early embryo expression programs involve compact genes with short introns and exons [20].

An emphasis of our analysis was the examination of the repetitive elements that are embedded in the introns and exons of genes whose abundance changes between the states we studied. Repetitive elements account for large portions of the human and mouse genomes and have been shown to have a multitude of roles in gene regulation and evolution [62–66]. Alu in primates and B elements in rodents offer a characteristic such example.

Despite the fact that Alu and B elements evolved independently after the primate-rodent split, we showed previously that they have significant genomic overlap with the intronic regions of genes belonging to the same pathways (such as translation, DNA replication, and RNA splicing) in both organisms [38]. Similar genomic links were also shown in subsequent work [67]. Notably, the very pathways that were highlighted in our earlier DNA-based, *genome-centric* analysis [38] emerge from the RNA-based, *transcriptome-centric* analysis of the current study. This suggests that the genomic distribution and architectural details of genes is tightly coupled to the transcriptional programs in which the genes participate.

In fact, the current work together with our previous findings [38] strongly suggests that the genes that are active during the early embryo expression trajectories have characteristic composition (enriched or depleted in repetitive elements) and architecture (short or long introns and exons). It is worth noting that the bimodal preferences between genes expressed in the zygote and the blastocyst, respectively, are encountered in both human and mouse. Coupling this with the observation that the bimodal gene composition is shaped by the presence or absence of organism-specific repetitive elements (Alu in human/primates, B elements in mouse/rodents), it follows that the links among gene length, gene complexity, gene content, and biological process [15, 27, 68] are an evolutionary solution that has been arrived at independently by different lineages [38].

It is important to note a distinction between our work, which studies the repetitive-element content of expressed genes, and previous work that studied the abundance of independently transcribing, bona fide repetitive elements. Indeed, it was previously shown that the stem cell phenotype correlates with increased expression of transposable elements [33, 34, 37]. Theunissen et al. [36] experimentally demonstrated that the expression of transposable elements is a better predictor of stem cell state than protein-coding gene expression and can provide a robust descriptor of pluripotency in human preimplantation embryo. Similarly, Boroviak et al. [53] reported that the dynamics of transposable element expression can distinguish preimplantation developmental stages and that repetitive-element expression had common but also organism-specific characteristics when comparing mouse and primate embryos.

On the other hand, our study examined the repetitive-element content in the introns and exons of independently transcribing protein-coding genes whose abundance changes during the preimplantation stages or during differentiation. It is particularly notable that these two independent schemes arrived at congruent results. It thus follows that the transcription of repetitive elements and the parallel transcription of genes with specific repetitive-element content are coordinated processes. We conjecture that this coordination is aimed at leveraging the sequences that these two groups of transcripts share for regulatory purposes [3, 38, 43, 71]. We discuss this conjecture next.

Indeed, the *transcriptome-based* findings we described above are strongly concordant to our earlier *genome-based* findings that emerged from the study of pyknon motifs [40] in non-coding and in protein-coding DNA. At the time, we reasoned that pyknons may serve as points-of-contact to effect regulation *in trans* [38, 40] and as sources of short RNAs. Since then, several independent discoveries [48, 69, 70] provided extensive support for such a regulatory network [71] and the production of short RNAs [69, 72]. More recently, we also generated evidence in support of the *organism-specific* aspect of this pyknon-based regulation. Specifically, we showed that the pyknons that are embedded in two primate-specific long non-coding RNAs, N-BLR [43] and FLANC [49], are responsible for the regulatory effect of these RNAs on mRNA expression and on colon cancer survival.

One intriguing finding pertains to the enrichment of repetitive elements in the PluriNet signature. The gene set comprising PluriNet involves evolutionarily conserved genes [56]. However, their introns are enriched in primate- (Alu) and mouse-specific (B elements) repetitive elements (Fig. 5) [3]. One limitation of our study is the interchangeable use of the terms “proliferative” and “pluripotent.” The two states may be distinct, but the data that we analyzed here do not allow us to separate the two. When such data become available, or under different physiological contexts, it will be interesting to dissect the coupling of each state to the architectural patterns of the expressed genes.

We examined gene expression in three post-blastocyst developmental time points and identified expression changes with reference to the blastocyst (Fig. 6b–e). There was a consensus among the three cases that the increased expression of short genes in pre-implantation stages is followed by increasing expression of longer ones in post-implantation development. We note the variability observed in the H1 and H9 embryonic stem cell lines (Fig. 6c). This variability was not reflected at the pathway level (Supplemental Table S1). The discrepancies regarding intron length biases could be due to the inherent variable nature of culturing cell lines or the different sex of the H1 and H9 donors [73]. Another potential explanation could be subtle underlying differences between the H1 and H9 transcriptomes that could predispose cells, or subpopulations within the culture, to diverge during differentiation. Such dynamic transcriptional “states,” particularly transient ones as described by Shaffer et al. [74], cannot be captured by the bulk RNA microarray analyses that were carried out at the time.

We also observed that, with the exception of LINE/L1 elements, there is a coherent enrichment or depletion of repetitive elements in preimplantation development (Fig. 3). However, this enrichment is not evident during post-implantation growth where we find Alu and MIR element densities to have opposite patterns (Fig. 6b). MIR elements were previously associated with tissue-specific gene expression patterns [67] as well as tissue-specific enhancer activities and erythropoiesis [75]. MIR elements were also shown to act as insulators [44]. Our analysis found them to be the only family of repeats that is significantly over-represented in the introns of genes that are up-regulated as human embryogenesis progresses from the blastocyst stage (Fig. 6b). This suggests a central role for MIR in shaping development patterns. Intriguingly, evidence at the level of the epigenome suggests the presence of a tissue-specific methylation profile of transposable elements that correlates with the tissue-specific expression patterns of adjacent protein-coding genes [76]. These data paint a picture where there is a complex interplay among factors promoting differentiation and establishing tissue identity, inter- and intra-genic regulatory regions, and repetitive-element distribution. The example of MIR elements involved in enhancer and insulator function suggests that repetitive elements could be driving transcription factor binding patterns during development [77]. Indeed, Rohrmoser et al. [78] used normal hematopoietic and cancer cell lines to show that ZNF768 binds to MIR elements and interacts with nuclear factors regulating gene expression.

Transcription factors were also flagged by our study as being important for tissue classification (Additional file 2: Supplemental Table S1). This is concordant with previous findings showing transcription factors to have profound roles in shaping tissue identity [79]. Kunarso et al. [80] provided further evidence on the involvement of transposons in transcription factor binding by utilizing embryonic stem cells from humans and mice and examining the binding patterns of important stem cell regulators, including the pluripotency-maintaining transcription factors OCT4 and NANOG. Analogously, we showed that a transposon embedded in Nanog’s mRNA is targeted by microRNA (miRNA) miR-134 [45, 46]. Within this context, it is an open question as to whether tissue specificity emerges from transcription factors and miRNAs that are guided by sequence motifs and binding sites as well as by the target gene’s architecture.

One further implication of our results is the emerging interplay between short non-coding RNAs, long non-coding RNAs, and messenger RNAs that contain repetitive elements. As these recurring pyknons are embedded in genes of specific architecture, as well as in non-coding RNAs that are transcribed independently, the common sequences could serve as contact points for miRNAs [40]. They can also serve as contact points for tRNA-derived fragments (tRFs) [81, 82], give rise to short regulatory RNAs through the formation of double-stranded RNA [3], provide decoy sequences for miRNAs or RNA binding proteins [43, 83], or serve purposes that are not currently understood in order to guide the transitions between pluripotency states. In fact, as mentioned above, a B element serves as a substrate for miR-134 during mouse embryonic stem cell differentiation [45, 46]. It is also worth recalling that tRNA fragments and piRNAs have already been directly linked with stemness [84–86], and pyknons have been linked with piRNAs [3, 40, 71].

## Conclusions

By analyzing gene expression datasets and signatures, we were able to uncover notable properties of the architecture and composition of genes involved in proliferation/pluripotency and differentiation. We found genes involved in proliferation/pluripotency to be shorter and denser in repetitive elements, particularly in Alu elements, while genes involved in differentiation and tissue identity to be longer and Alu-sparser. Our findings suggest that repetitive-element sequences are strongly coupled to the underlying events and potentially make major, non-random, and organism-specific contributions to gene expression changes across cell states.

## Materials and methods

### Definitions

We define the “exonic region” of a gene as union of its exons. We define the “exonic length” of a gene as the length of its exonic region expressed in number of base pairs. We define a gene’s “intronic region” as what remains after subtracting its exonic region from the gene’s genomic span. We define the “intronic length” of a gene as the length of its intronic region. We define the “exonic content” of a gene as the fraction of the gene’s genomic span that is taken up by the gene’s exons. We also refer to a gene’s exonic content as the “gene compactness” or a gene’s “exonic density.” We define a genomic region’s “density in repetitive element family X” as the fraction of the region’s span that is taken up by repetitive elements belonging to family X. Depending on the task, we can distinguish between the “density in repetitive element family X” of introns and of exons.

### Data acquisition and processing

This study is based on publicly available datasets. From the study of Xie et al. [50], we downloaded CEL files from GEO (GSE18290). We processed the blastocyst and 1-cell embryo datasets with the *affy* package in R [87] and normalized with the robust multi-average (RMA) algorithm with default parameters but without quantile normalization. We sub-selected among protein-coding genes based on expression and removed from further consideration the 25% with lowest mean expression. This was done separately for human and mouse datasets. A total of 13,736 human genes and 10,238 mouse genes survived this filtering step.

From the study of Kim et al. [58], we downloaded the CEL files from GEO (GSE54186) and processed them as we did for the data of Xie et al.

From the study of Petropoulos et al. [51], the RPKM-normalized dataset was downloaded from ArrayExpress (E-MTAB-3929). For these analyses, we considered the embryonic days 3 (8-cell embryo) and 7 (late blastocyst). For each embryo, we combined the expression of the single cells into one vector, computing the average expression of each gene per embryo. We kept the 50% most expressed genes (a total of 13,034 genes), to work with approximately the same number of genes as in the microarray studies. For the three lineages, we used the 100 maintained lineage-specific genes as reported Supplemental Table S2 of Petropoulos et al.

From the study of Sharma et al. [52], we used the data contained in the “Additional Data Table S7.” Our analyses used the 11,076 genes reported by that study for the blastocyst and 2-cell expression profiles.

From the study of Yi et al. [57], we examined the 2280 genes that were reported in Supplemental Table S3 and kept those with decreasing or increasing expression.

The PluriNet [56] signature was obtained from MSigDB [88].

The RPKM data from the study of Cardoso-Moreira et al. [59] were downloaded from ArrayExpress (E-MTAB-6798 for mouse and E-MTAB-6814 for human) and we kept genes with an average expression of more than 2 RPKM across samples.

The v7 TPM-normalized GTEx dataset was downloaded from the GTEx portal (https://www.gtexportal.org/home/datasets) on June 29, 2018. The whole dataset comprised 56,202 genes. After excluding samples with severe autolysis score, we assigned each one to its corresponding tissue type and excluded tissues with 40 or fewer samples. This resulted in 27 tissues and a total of 11,564 samples. Then, for each sample, we kept the genes with > 2 FPKM and binarized the profile by considering as “expressed” the top 50% most expressed genes in that sample. The average expression threshold was 13 FPKM (Additional file 5: Supplemental Figure S4). Genes considered as “not expressed” in fewer than 50% of the samples within all tissues were filtered out of the analysis. Also, genes found “expressed” in more than 90% of the samples within all tissues were labeled as “widely expressed” and did not participate in the machine learning.

Homeobox-containing (HOX) genes were downloaded from *www.genenames.org* on August 25, 2019.

### Genomic computations

For consistency with the obtained microarray data, we used the GRCh37 assembly of the human genome and Rel. 75 of ENSEMBL. For mouse, we used the GRCm38 assembly of the mouse genome and Rel. 94 of ENSEMBL [89]. We identified the mouse orthologues of human genes with the help of the BioMart tool. Our analyses include only protein-coding genes. Only for the GTEx genes, as they were annotated on the more recent version of the human genome, we used the GRCh38 assembly with Rel. 94 of ENSEMBL.

### Repetitive elements

Information about repetitive elements was obtained from the RepeatMasker tables (http://www.repeatmasker.org) for GRCh37, GRCh38, and GRCm38, respectively. We computed overlaps with exons and introns at the level of repetitive class/family (Additional file 3: Supplemental Table S2) and excluded repeats with low confidence (marked with a question mark), simple, and low complexity repeats.

Pyknon sequences [40] were searched in the exonic space as well as against the human genome (GRCh37) using an exhaustive brute force search. Then, the genomic coordinates of where pyknons exist were intersected with RepeatMasker entries. For a specific gene set (e.g., up-regulated in blastocyst as compared to early embryo), all the pyknons within the respective mRNAs were extracted and all genomic coordinates of those pyknons were found. We then counted how many of the pyknons overlapped with each RepeatMasker family, e.g., SINE/Alu elements. We note that there were multiple instances where one pyknon could be found in more than one families.

To normalize for gene length, we counted how many of the unique pyknons of the Venn diagram of Fig. 4a appear in each gene. We divided this number by the exonic length of each gene and normalized per 10,000 base pairs.

### Statistical analyses, machine learning, and visualization

For the data of Xie et al. [50], Petropoulos et al. [51], and Sharma et al. [52], we used significance analysis of microarrays (SAM) to calculate differentially abundant genes [90] with 5000 permutations, and a false discovery rate (FDR) of 5%. The study of Kim et al. [58] did not include adequate samples for statistical analyses, and we only considered genes whose expression changed between undifferentiated and differentiated cells by at least twofold.

As an approximation of the genes transcribed de novo following zygotic genome activation (ZGA), we used the samples from the Xie et al. study. Based on information from the literature [54], we approximated ZGA in humans by identifying the up-regulated genes in 8-cell embryos as compared to the immediately previous stage, i.e., the 4-cell embryos. For mouse embryo, we compared the 4-cell embryos with 2-cell embryos. Comparisons were done with SAM. For the comparison of Xie et al. and Cardoso-Moreira et al. [59], we ranked-normalized the expression per sample, with the gene of highest expression being ranked as 1. Then, we performed SAM between the blastocyst stage as reported in Xie et al. and the two earliest developmental time points per tissue as reported in Cardoso-Moreira et al., i.e., 4 weeks post-conception (wpc) and 5 wpc for humans and E10 and E11 for mouse. We chose two time points for the latter study to include more samples for increased statistical power. We performed SAM on the rank-normalized dataset (FDR < 1%). This methodology allowed us to identify genes whose rank changed in the tissues during embryogenesis as compared to the blastocyst (Additional file 2: Supplemental Table S1).

In order to identify a tissue-specific gene signature, we employed the methodology we developed previously [61]. We applied this approach to the GTEx cohort and analyzed 11,564 RNA-sequencing datasets from 27 human tissues. Filtering (see the “Data acquisition and processing” section) left us with a total of 15,054 expressed genes across all samples. The median number of expressed genes per sample was 5634 (Additional file 1: Supplemental Figure S4A; Additional file 5: Supplemental Table S4). During the filtering process, we also identified and excluded 1340 genes that were widely expressed and, thus, could not possibly be part of a tissue-specific signature. We used the binarized dataset to train a multi-class support vector machine (SVM) model of linear kernel with 10-fold cross-validation. The SVM algorithm identifies the optimal hyperplane separating two tissues. By performing all pairwise comparisons and by using a voting algorithm, the model is able to assign a newly seen sample in one of the tissues with a probability score. If the probability for the most-voted tissue is lower than 0.5, then we assign the sample to an “Other” class. The resulting SVM model was able to correctly assign samples to their tissue of origin with an average accuracy of 99% and an average FDR of 0.004 (Additional file 1: Supplemental Figure S4B). We extracted the variable importance (VI) score for each gene as the average of the squared weights across all pairwise comparisons [61]. The genes with the highest VI scores (Additional files 2 and 5: Supplemental Tables S1 and S4) were able to classify correctly the 27 different tissues (Fig. 7a). This tissue-specific signature comprised a total of 1505 distinct genes. The SVM model was developed in R with the *svm* function of the *e1071* package.

The background gene set was specific for each study. For Xie et al. [50], Petropoulos et al. [51], and Sharma et al. [52] and the integrative study of Xie et al. and Cardoso-Moreira et al., the background comprised all of the genes that entered the differential abundance analysis (SAM on abundance or ranking). For Yi et al. [57], we used as background the 2280 genes reported by that study. For PluriNet, the background comprised all protein-coding human genes. For the evaluation of the lineage-specific genes of Petropoulos et al. [51], we used all human protein-coding genes. The background for the widely expressed genes as identified in the GTEx study was all human protein-coding genes; the background for the tissue-specific gene signature was the genes included in the machine learning.

Kolmogorov-Smirnov tests were used to evaluate statistically significant shifts in the cumulative distributions of exon and intron length and of exonic content of the considered gene set as compared to the background distribution.

To evaluate the statistical significance of overlap with repetitive elements, we carried out Monte Carlo simulations with 10,000 iterations. During each iteration, we randomly chose genes from the background equal in number to the genes being studied: for each such random choice, we computed the exons’ and introns’ average “density in repetitive family X,” respectively. X ranged over all repetitive families. Upon completion of the 10,000 iterations, we constructed a distribution of “expected” density values that we then used to calculate the *Z*-score of the “observed” values. Density values were calculated separately for each family of repetitive elements. We consider values of absolute *Z*-score ≥ 2 to represent a statistically significant enrichment (positive *Z*-scores) or depletion (negative *Z*-scores). We also conducted Kolmogorov-Smirnov tests to examine whether the cumulative distribution in repetitive-element density is different from the background population. Resulting *P* values were corrected to FDR, and *Z*-scores that were associated with an FDR larger than 5% were not considered significant. The actual values are included in Additional file 4: Supplemental Table S3.

To visualize our findings, we plotted differences from the background cumulative distribution (see legend of Fig. 2). To this end, we represented the background by the horizontal axis *Y* = 0. For a given choice of *X* (= intron length, exon length, or exonic content), data points above this horizontal axis signify an increase with regard to background, i.e., a shift towards genes with longer introns, longer exons, or higher exon density. Data points below the horizontal axis signify the opposite.

## Supplementary Information

**Additional file 1: Supplemental Figure S1.** Controlling for the background gene set. **Supplemental Figure S2.** Zygotic genome activation. **Supplemental Figure S3.** Differentially ranked genes in embryonic tissues compared to the blastocyst. **Supplemental Figure S4.** Binarizing expression profiles and Support Vector Machines (SVMs). **Supplemental Figure S5.** Properties of HOX genes.

**Additional file 2: Supplemental Table S1.** Gene sets and enriched pathways.

**Additional file 3: Supplemental Table S2.** Gene characteristics and architecture.

**Additional file 4: Supplemental Table S3.** Repetitive elements enrichments/depletions in the analyzed gene sets.

**Additional file 5: Supplemental Table S4.** Binary dataset and SVM model VI scores.

## Data Availability

The datasets analyzed during the current study are available in the GEO repository (GSE18290, GSE54186), ArrayExpress (E-MTAB-3929, E-MTAB-6798, E-MTAB-6814), or GTEx portal. Data generated or analyzed during this study are also included in this published article and its supplementary information files.
