# Supplementary material for: The transcriptional trajectories of pluripotency and differentiation comprise genes with antithetical architecture and repetitive-element content
Source: BMC Biol. 2021 Mar 25;19:60. doi: 10.1186/s12915-020-00928-8 (PMC7995781; doi:10.1186/s12915-020-00928-8)
Supplement: Supplementary file 1 — Additional file 1: Supplemental Figure S1. Controlling for the background gene set. Supplemental Figure S2. Zygotic genome activation. Supplemental Figure S3. Differentially ranked genes in embryonic tissues compared to the blastocyst. Supplemental Figure S4. Binarizing expression profiles and Support Vector Machines (SVMs). Supplemental Figure S5. Properties of HOX genes. [file 12915_2020_928_MOESM1_ESM.docx]

**The Transcriptional Trajectories of Pluripotency and Differentiation Comprise Genes with Antithetical Architecture and Repetitive-element Content**

**Supplemental Figures**

**Supplemental Figure S1: Controlling for the background gene set.** (A-B) Venn diagrams comparing the background gene sets of the two datasets per organism. A total of 16,551 protein-coding genes were included in the human datasets (A) and 13,911 in mouse (B) with 10,217 and 7,373, respectively, common in both of the analyzed datasets. In the context of all protein-coding genes in the respective organisms, these overlaps are statistically significant (P<10^-3^; Hypergeometric test), indicating a considerable reproducibility in the two gene expression studies. (C-D) Difference of the cumulative distribution of exonic length (top), intronic length (middle) and exon content (bottom) of upregulated (orange curve) and downregulated (blue curve) genes from the respective distribution of all protein-coding genes in human (C) and mouse (D). Gray lines represent the background cumulative distributions projected on the secondary Y axis. Vertical lines were drawn at the median value of each gen eset. Asterisks indicate a statistically significant difference from the background (black) curve (P<10^-4^; Kolmogorov-Smirnov test).

**Supplemental Figure S2: Zygotic genome activation.** (A-C) Difference of the cumulative distribution of exonic length (A), intronic length (B) and exon content (C) of ZGA-activated genes from the respective background distribution in human (light green) and mouse (dark green). Asterisks indicate statistical significance (P<10^-4^; Kolmogorov-Smirnov test). Asterisks indicate a statistically significant difference from the background (black) curve (P<10^-4^; Kolmogorov-Smirnov test). (D-E) Venn diagrams showing the overlap of ZGA-activated genes (blue circles) with the genes found upregulated when comparing the blastocyst and the 1-cell embryo (red curves). In the context of the background genes for these studies, the overlap is statistically significant (P<10^-4^; Hypergeometric test). The results in these panels come from analyzing the Xie *et al*. human and mouse datasets.

**
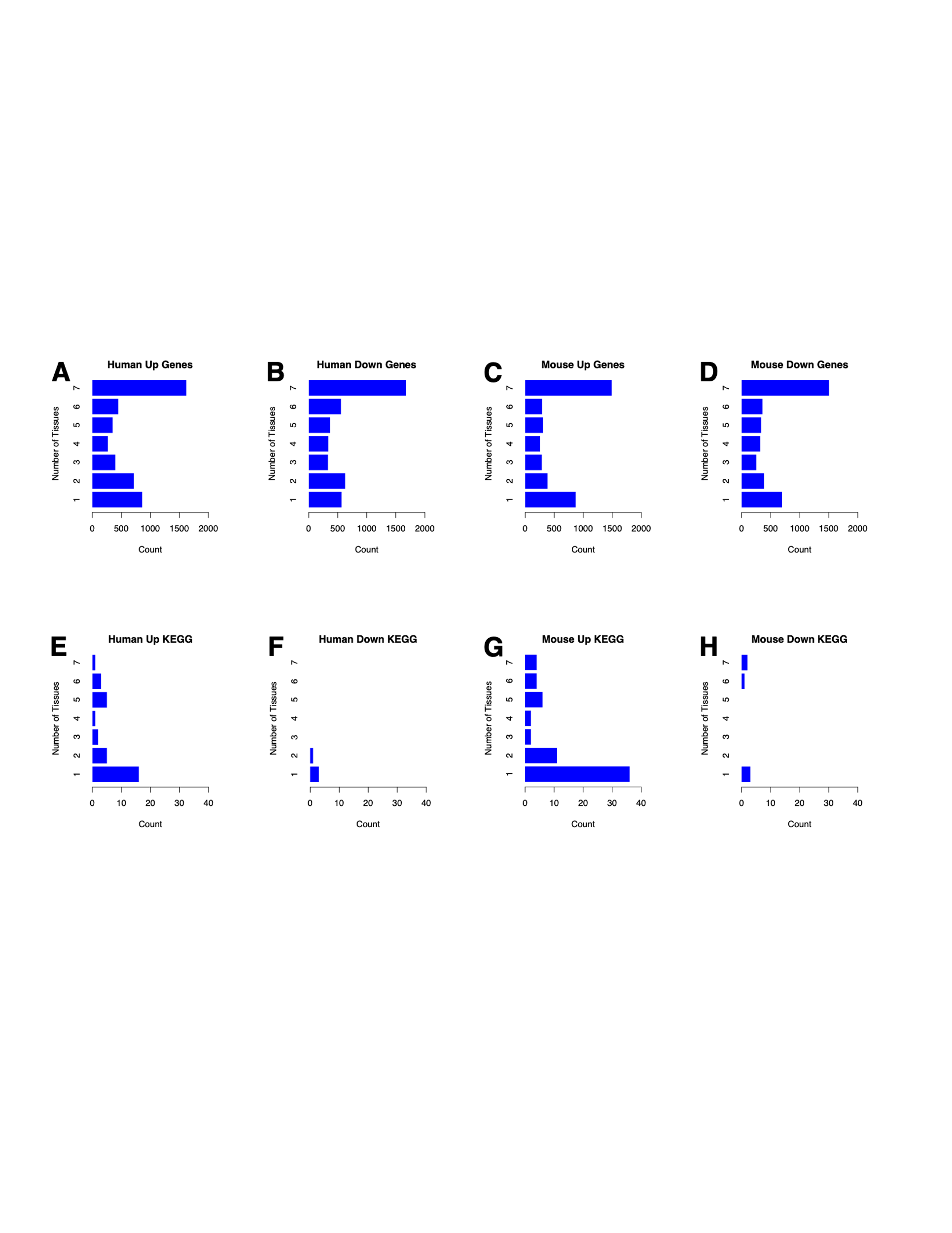
**

**Supplemental Figure S3: Differentially ranked genes in embryonic tissues compared to the blastocyst.** (A-D) Barplots showing how many (X axis) differentially ranked genes are common among tissues (numbers across the Y axis) for human (A-B) and mouse (C-D). (E-H) Barplots showing how many (X axis) KEGG pathways are commonly enriched among tissues (numbers across the Y axis) for human (E-F) and mouse (G-H). Up: upregulated genes or pathways enriched in the upregulated genes; Down: downregulated genes or pathways enriched in the downregulated genes. The lists with the genes and pathways can be found in Supplemental Table S1.

**Supplemental Figure S4: Binarizing expression profiles and Support Vector Machines (SVMs).** (A) Barplot showing the FPKM threshold for each sample, above of which the genes were marked as ‘expressed’. Each data point represents an RNA-sequencing sample and samples are grouped based on tissue. (B) Classification of the GTEx samples using the SVM model trained on the 15,054 genes. The rows of the heatmap show the original tissue of origin and the columns the predicted tissue type. The color shade of each cells indicates the percentage of samples that were of the ‘original’ respective tissue and were ‘predicted’ to be the respective tissue type. The ‘Other’ category captures samples with low prediction probability. The high percentages on the diagonal indicate the high accuracy of the model. As the SVM was run with 10-fold cross-validation, the heatmap indicates the average of 10 runs. From this analyses, we extracted the genes with the highest variable importance (VI) scores, i.e. the ones that have the highest absolute weights in classifying the samples, and re-trained the model that is presented on Figure 5A.

**Supplemental Figure S5: Properties of HOX genes.** Exon (A) and intron (B) length biases in HOX genes. The red curve shows the difference between the cumulative distribution of the HOX genes from the background distribution of all human protein-coding genes. Asterisks indicate a statistically significant difference (P-value < 10-4; Kolmogorov-Smirnov test). (C) Heatmap of enrichment and depletion scores of the repetitive density in the intronic regions of the HOX genes in human. The HOX genes deviate from the observation that genes establishing tissue identity are longer and sparse in repetitive-elements.
